# Joint population pharmacokinetic modeling of venlafaxine and O-desmethyl venlafaxine in healthy volunteers and patients to evaluate the impact of morbidity and concomitant medication

**DOI:** 10.3389/fphar.2022.978202

**Published:** 2022-12-08

**Authors:** Zhanzhang Wang, Lu Li, Shanqing Huang, Xipei Wang, Shujing Liu, Xiaolin Li, Wan Kong, Xiaojia Ni, Ming Zhang, Shanshan Huang, Yaqian Tan, Yuguan Wen, Dewei Shang

**Affiliations:** ^1^ Department of Pharmacy, The Affiliated Brain Hospital of Guangzhou Medical University, Guangzhou, China; ^2^ Guangdong Engineering Technology Research Center for Translational Medicine of Mental Disorders, Guangzhou, China; ^3^ School of Pharmacy, Guangzhou Medical University, Guangzhou, China; ^4^ Medical Research Center, Guangdong Province People’s Hospital, Guangdong Academy of Medical Sciences, Cardiovascular Institute, Guangzhou, China

**Keywords:** venlafaxine, O-desmethyl venlafaxine, joint population pharmacokinetic model, pharmacokinetics, precision medication

## Abstract

**Introduction:** Venlafaxine (VEN) is a widely used dual selective serotonin/noradrenaline reuptake inhibitor indicated for depression and anxiety. It undergoes first-pass metabolism to its active metabolite, O-desmethyl venlafaxine (ODV). The aim of the present study was to develop a joint population pharmacokinetic (PPK) model to characterize their pharmacokinetic characters simultaneously.

**Methods:** Plasma concentrations with demographic and clinical data were derived from a bioequivalence study in 24 healthy subjects and a naturalistic TDM setting containing 127 psychiatric patients. A parent-metabolite PPK modeling was performed with NONMEM software using a non-linear mixed effect modeling approach. Goodness of fit plots and normalized prediction distribution error method were used for model validation.

**Results and conclusion:** Concentrations of VEN and ODV were well described with a one-compartment model incorporating first-pass metabolism. The first-pass metabolism was modeled as a first-order conversion. The morbid state and concomitant amisulpride were identified as two significant covariates affecting the clearance of VEN and ODV, which may account for some of the variations in exposure. This model may contribute to the precision medication in clinical practice and may inspire other drugs with pre-system metabolism.

## 1 Introduction

Venlafaxine (VEN) is a dual selective serotonin/noradrenaline reuptake inhibitor approved by the FDA for the treatment of depression, social anxiety disorder and cataplexy (Singh and Saadabadi, 2022). The recommended daily dose of VEN is 75–225 mg and the main adverse events with regard to this drug are drug ineffective, toxicity, suicide, drug interaction and nausea (FDA, 2021).

VEN is well absorbed. The absolute bioavailability of VEN is 45% for extended-release formulation and approximately 12.6% for regular-release capsule (Ellingrod and Perry, 1994). VEN and ODV are minimally bound at therapeutic concentrations to plasma proteins (27% and 30%, respectively); therefore, they are not likely to increase the free concentrations of other drugs with high protein binding (Wyeth Pharmaceuticals LLC, 2021). VEN undergoes significant first-pass metabolism in the intestine and liver to one major active metabolite, O-desmethyl venlafaxine (also known as desvenlafaxine, ODV) (Taft et al., 1997), and two minor less active metabolites, N-desmethyl venlafaxine and N,O-didesmethyl venlafaxine by cytochrome P-450 (CYP) isozymes. The CYP2D6, and to a less extent CYP2C19, plays a dominant role in VEN and ODV metabolism (Klamerus et al., 1992). Several studies have investigated the pharmacokinetic profiles of VEN and ODV in healthy subjects. The mean half-life varied from 2 to 13 h for VEN, and from 10 to 19 h for ODV, respectively (Klamerus et al., 1992; Bhatt et al., 2005; Wei et al., 2007; Reddy et al., 2013). The mean absorption times were 1.4–1.6 h for VEN and 2.2 h for ODV (Klamerus et al., 1992). The apparent clearance of VEN and ODV were 1.3 ± 0.6 L/h/kg and 0.4 ± 0.2 L/h/kg, and the apparent (steady-state) volume of distribution was 7.5 ± 3.7 L/kg for VEN and 5.7 ± 1.8 L/kg for ODV, respectively (Wyeth Pharmaceuticals LLC, 2021).

A number of studies have indicated significant intra- and inter-individual pharmacokinetic variability for VEN and ODV. The influences of sex, age, gene polymorphism (CYP2D6, CYP2C19) and comedications (e.g., valproic acid, doxepin, trimipramine, quetiapine) have been previously reported (Klamerus et al., 1996; McAlpine et al., 2011; Paulzen et al., 2018a; Paulzen et al., 2018b; Lin et al., 2018; Kowalewski et al., 2019; Wang et al., 2020). However, studies exploring these effects on dose and concentration have shown inconsistent results and mixed effects. Therapeutic drug monitoring (TDM) as an auxiliary tool for precision medication has been recommended for routine management with VEN (recommendation level 2 (Hiemke et al., 2018)). The therapeutic reference range for the sum of VEN and ODV is 100–400 ng/ml, with a laboratory alert level of 800 ng/ml (Hiemke et al., 2018). Considering the half-life and dosing regimen, it generally requires 3–5 days to reach a steady state. Thereafter, dose increases should be made based on TDM results and upward titration usually needs 2 weeks or more.

Population pharmacokinetic (PPK) modeling is a robust tool for precision medication (Li et al., 2012). Compared with classic pharmacokinetic modeling, which needs a complete set of concentrations at each time point, PPK obtains pharmacokinetic parameters from sparse or intensive concentration data. It also enables quantitative evaluation of mixed effects of potential covariates and characterizes the inter-individual and intra-individual variabilities. Concentrations upon various conditions could be predicted with few restrictions. PPK has been successfully applied to a variety of medications. However, as far as we know, only two PPK models for VEN in non-English languages, and one PPK model for ODV, have been published. Xie et al. (2020) reported a PPK model for VEN in Chinese healthy subjects. The typical value was 104 L/h for CL/F and 78.8 L for V/F, with no covariate identified (Xie et al., 2020). Chen et al. (2014) established a PPK model for Chinese patients with depression using a fixed absorption rate (0.08 h^−1^). The CL/F for VEN was 83.7 L/h and the V/F was 343 L (Chen et al., 2014). Body weight was found to affect the distribution volume, and creatinine showed an influence on clearance (Chen et al., 2014). Nichols et al. (2018) developed a PPK model for ODV using fixed values concerning absorption. The subjects were administrated with the compound ODV (Nichols et al., 2018). The population typical values for CL/F and V/F were 19.53 L/h and 282 L for ODV, respectively. Pharmacokinetic parameters of ODV were similar between MDD patients and healthy subjects, and between people from different populations (Nichols et al., 2018). As shown in the PPK model for ODV, CLCR, WT, age group, sex, multiple dosing and alkaline phosphatase levels were significant factors on the CL/F; and WT, sex, multiple dosing and food were significant factors affecting V/F (Nichols et al., 2018). However, none of these models include the two compounds simultaneously, limiting their applications for clinical practice.

Therefore, this study aims to investigate the pharmacokinetic characters of both VEN and ODV simultaneously, using PPK modeling approaches. For this purpose, a complete data set of pharmacokinetic study in healthy Chinese subjects and routine sparse TDM data from patients with mental illness were analyzed. A joint PPK model for VEN and ODV was developed without fixed values of absorption rate constant or first-pass metabolism fraction and sources of variability contributing to clearance and distribution volume were explored.

## 2 Methods

### 2.1 Study design and analytical method

#### 2.1.1 Study 1

This study was a single-center, single-dose, randomized, crossover bioequivalence study in 24 healthy male adult volunteers. VEN hydrochloride capsules of 25 mg were supplied from two manufacturers: Minsheng Pharmaceutical Group Co., Ltd. (China) and Chengdu Kanghong Pharmaceutical Group Co., Ltd. (China). After fasting for at least 12 h, volunteers were administrated with 50 mg VEN hydrochloride. There was a washout period of 7 days between each two consecutive study drug administrations. Fifteen venous blood samples (4 ml) were drawn from each individual pre-dose and at 0.5, 1, 1.5, 2, 2.5, 3, 3.5, 4, 6, 8, 10, 12, 24, and 36 h post-dose in each period. Plasma samples were centrifugated at 3,500 rpm for 10 min and stored in polypropylene tubes at −20°C.

VEN concentrations were measured with liquid chromatography-tandem mass spectrometry (LC-MS/MS) (Ni et al., 2011). A set of VEN calibrators at seven levels (0.2–200 ng/ml) and quality controls (QCs) at 0.5, 100, and 150 ng/ml were prepared in human plasma. The linearity was acceptable (weighting coefficient = 1/x^2^, correlation coefficients = 0.9984). Limits of precision and accuracy for calibrators and QCs were ±20% at the lower limit of quantification (LLOQ) and ±15% at other levels. The matrix effect was consistent between QCs. No endogenous interferent was found at retention times of VEN and the internal standard of tramadol. Data below the LLOQ at the absorption phase was assigned as zero, while excluded from statistics at the elimination phase.

#### 2.1.2 Study 2

This was a retrospective study based on real-world TDM data and the hospital information system of the Affiliated Brain Hospital of Guangzhou Medical University. Most values were trough concentrations taken at 6–7 am before medication. The dosing regimens included once daily, once a night, twice or three times a day, and other special intervals. Patients hospitalized from March 2018 to February 2021 taking VEN as a monotherapy or as combination therapy were enrolled. Demographic data (age, weight, and sex), smoking and drinking history, duration of current therapy, concomitant drug therapy, comorbidities, and VEN dosing regimens were determined throughout the study. Biochemical parameters, including serum alanine transaminase (ALT), aspartate aminotransferase (AST), creatinine (CR) and blood urea nitrogen (BUN) were also obtained.

The simultaneous determination of VEN and ODV was conducted by LC-MS/MS (Xie et al., 2019). The linearity ranges were 4–400 ng/ml for VEN, and 20–2,000 ng/ml for ODV. Analytes were extracted by protein precipitation using acetonitrile. Deuterated internal standards of VEN-*d6* and ODV-*d6* were used. The linearity, sensitivity, matrix effect, extraction efficiency, accuracy, precision and stability were validated and acceptable according to guidelines by Chinese Pharmacopoeia (Castberg et al., 2017). Data beyond quantitative ranges was labeled and excluded in this study.

### 2.2 Pharmacokinetic modeling

**FIGURE 1:**
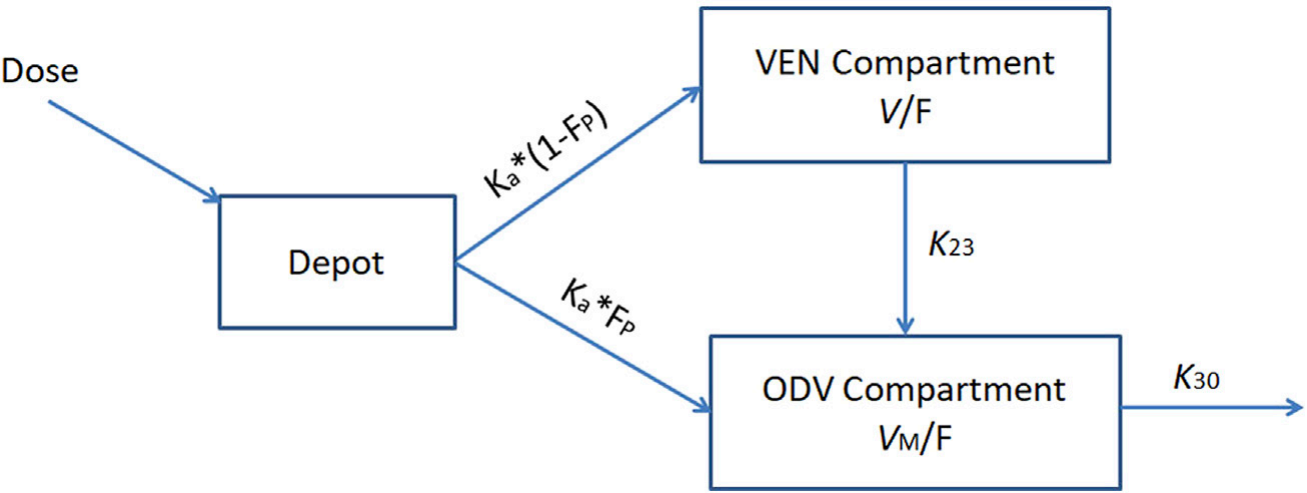
Population pharmacokinetic model structure for VEN and ODV. *K*_a_, absorption rate constant, *K*_23_, rate constant for the conversion of VEN into ODV; *K*_30_, elimination rate constant for ODV; *V*/F, apparent volume of distribution for VEN; *V*_M_/F, apparent volume of distribution for ODV; F_P_, fraction of VEN converted into ODV before entering the blood circulation.

Population analyses were performed using non-linear mixed effects models (NONMEM 7.3, ICON Development Solutions, Hanover, MD, United States) with Perl speaks NONMEM (PsN version 4.2.0) as an interface. Pirana (version 2.9.7) and R (version 4.0.3) were applied for processing and visualization. Population pharmacokinetic data analyses were performed between March 2021 and April 2022. Based on the data characteristics, as TDM samples were mostly drawn in the morning before dosing, the concentrations were analyzed according to a one-compartment pharmacokinetic model. Parameters of the structural model to be estimated included the clearance (CL/F) and the volume of distribution (*V*/F) of VEN; the clearance (CL_M_/F) and the volume of distribution (*V*_M_/F) of ODV; and the fraction of the absorbed dose of VEN converted into ODV in the gut (F_P_). Model parameters were estimated using the first-order conditional estimation method with interaction (FOCEI). The plasma concentration-time profiles for VEN and ODV were described by a base structural model using the subroutine ADVAN6 with a precision of integration solution set to six significant digits (TOL = 6). The final model structure for VEN and its active metabolite, ODV, is depicted in Figure 1. Key features of this joint population model are as follows: 1) pre-systemic metabolism of VEN and conversion to ODV in the gut, 2) one-compartmental disposition for both VEN and ODV. In other words, an amount of active constituents can enter the central compartment after oral dosing with an absorption rate constant (K_a_). A proportion (FP) of the parent drug (VEN) was transformed into its active metabolite (ODV) *via* first-pass effect. VEN in the central compartment was then transformed to ODV by a first-order process (*k*_23_) and eliminated from the system (CL/F). Meanwhile, ODV was cleared from the system (CL_M_/F) with an elimination rate constant (*K*_30_).

The interindividual variability (IIV) of the PK parameters was assumed to be log-normally distributed, and an exponential model was used to access the IIV: P_j_ = P_TV_ × e^η^_p_, where P_j_ is the predicted parameter for the individual j, P_TV_ is the population typical value of the parameter, and η_p_ is the difference in the estimated parameter for the *j*th subject, which was normally distributed with a mean of zero and a variance of ω_p_^2^.

For the parent and metabolite, the residual error was modelled using a proportional error model as follows: *y*_ij_ = *y*_ij_ *’*× (1+*ε*_ij_), where y_ij_ is the *j*th observation in the *i*th individual, y_ij_’ is the model’s predicted value; and ε_ij_ is the normally distributed random errors with mean values of zero.

Health status, VEN formulation, sex, age, body weight, smoking history, drinking habit, concomitant medications (valproic acid, quetiapine, clozapine, olanzapine, risperidone and amisulpride) were considered potential variables for pharmacokinetic parameters. The full model was built by stepwise forward inclusion. Each covariate was considered statistically significant if the objective function value (OFV) from basic model decreased more than 6.63 (chi-square, *p* < 0.01, df = 1) after introducing a new covariate. Both continuous covariates (e.g., age and weight) and discrete covariates (e.g., sex, smoking status, and concomitant medications) were introduced into each parameter in a stepwise fashion. Afterward, the covariates were removed from the full model independently. A covariate was retained in the model if eliminating the covariate resulted in a rise of OFV greater than 10.83 (*p* < 0.001). The model was selected according to the reduction in the OFV value, goodness-of-fit plots, reductions in the IIV of structure model parameters, residual error, robust model parameter estimation, and model stability.

### 2.3 Model evaluation

The goodness-of-fit (GOF) plots (Tanaudommongkon et al., 2022) and normalized prediction distribution error (NPDE) (Li et al., 2012) method were utilized to evaluate the final models and parameter estimates. The GOF plots include individual (IPRED) and population (PRED) predictions vs. observed concentrations, and conditional weighted residuals (CWRES) vs. time after last dose and PRED (Hooker et al., 2007). The NPDE plots include the quantile-quantile plot (QQ plot) comparing the NPDE. *N* (0, 1) distribution, the NPDE histogram with the density of *N* (0, 1) overlaid, the NPDE plotted against time and predicted concentrations. The NPDE analysis was implemented by R-*npde* package (version 2.0).

## 3 Results

### 3.1 Participant characteristics

**FIGURE 2:**
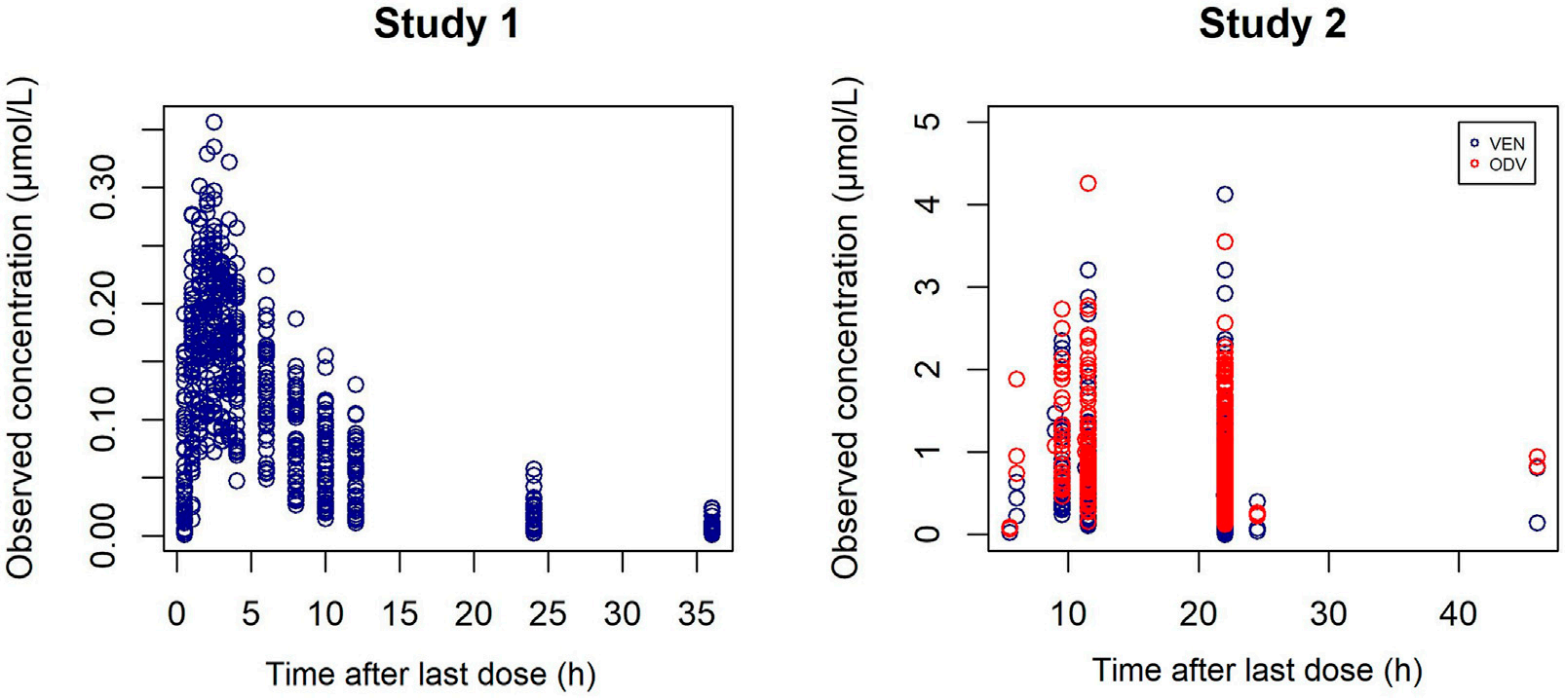
Individual C-t profiles of VEN (blue) and ODV (red) in study 1 (left) and study 2 (right).

The analysis included 664 concentrations from 24 healthy subjects and 804 concentrations from 127 psychiatric patients for modeling. A summary of the demographic and clinical characteristics of participants of both studies is shown in Table 1. The concentration points from each study are shown in Figure 2, which indicated wide IIV in the study group.

**TABLE 1:** Demographic and clinical summary of participants in the pooled analysis.

	Study 1	Study 2
Number of subjects	24	127
Number of concentrations	664	804
Sex, %	Male	Male 44.88%, Female 55.12%
Age, years (range)	23.13 ± 2.47 (18, 27)	37.86 ± 17.59 (14, 86)
Body weight, kg (range)	62.5 ± 6.9 (54, 80)	61.95 ± 11.02 (38, 93)
ALT, U/L (range)	21.67 ± 7.15 (7, 41)	22.92 ± 18.36 (3, 112)
AST, U/L (range)	19.21 ± 5.64 (15, 42)	22.22 ± 14.13 (9, 116)
BUN, mmol/L (range)	4.35 ± 0.97 (2.63, 5.84)	3.91 ± 1.36 (1.21, 8.61)
CR, μmol/L (range)	76.13 ± 12.41 (51, 106)	68.23 ± 16.15 (6.38, 129)
Smoke, %	—	7.87%
Drink, %	—	4.72%
Daily dose of venlafaxine, mg (range)	50	132.86 ± 64.20 (25, 300)
Formulation, %	Rapid release tablets	Sustained release tablets 83.62%, Rapid release tablets 16.38%
Valproic acid, %	—	18.16%
Quetiapine, %	—	23.38%
Clozapine, %	—	15.67%
Olanzapine, %	—	24.50%
Risperidone, %	—	13.18%
Amisulpride, %	—	5.47%

ALT, alaninetransaminase; AST, aspartate aminotransferase; BUN, blood urea nitrogen; CR, creatinine.

### 3.2 Population pharmacokinetic modeling

**FIGURE 3:**
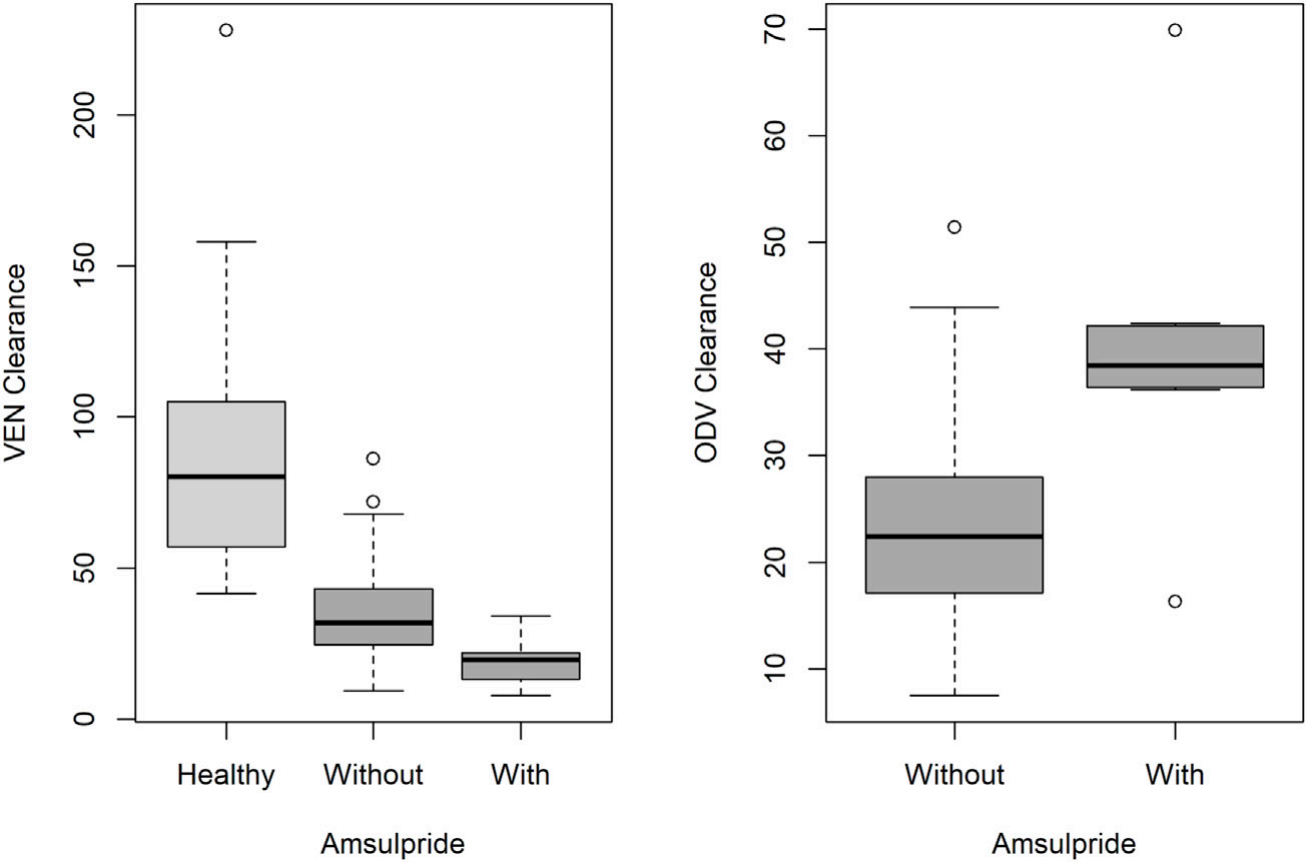
Boxplot with the median and interquartile range of the clearance of VEN (left) and ODV (right) according to health status and concomitant use of amisulpride. Box with light grey represents healthy subjects, and box with dark grey represents patients.

A one-compartment pharmacokinetic model with mixture error best described the concentration of VEN and ODV. The K_a_ for VEN was fixed at 0.63 h^−1^, according to the population typical value pre-estimated in a two-compartment pharmacokinetic model of VEN. Adding each covariate independently using stepwise forward inclusion improved the fit of the model. In the final model, health status was identified as a significant covariate for the clearance of VEN, and the concomitant use of amisulpride was identified as a significant covariate for the clearance of VEN and ODV. The clearance of VEN and ODV grouping with covariates is displayed in Figure 3.

The population-predicted clearance in healthy Chinese subjects was 81 L/h for VEN and 22 L/h for ODV, and the population-predicted volumes of distribution for VEN and ODV were 628 L and 238 L, respectively. By contrast, in psychiatric patients, the population-predicted clearance of VEN was 31 L/h. Morbidity state was associated with a decrease in the clearance of VEN by 61.7% (*p* < 0.001). The clearance of VEN and ODV in patients comedicated with amisulpride was 39.2% lower (*p* < 0.001) and 59.3% higher (*p* < 0.001) than in patients without amisulpride. Other covariates such as formulation, comedications of clozapine and quetiapine did not significantly influence the PK parameters of VEN and ODV. The final population PK parameters are summarized in Table 2.

**TABLE 2:** Pharmacokinetic parameters for the joint PPK model of VEN and ODV.

Parameters	Parameter estimates	RSE%
CL/F, L/h	80.9	9.7%
*V*/F, L	628	5.8%
CL_M_/F, L/h	22.1	6.6%
*V*_M_/F, L	238	33.1%
K_a_, 1/h	0.63	--
F_P_	0.048	18.3%
θ_morbid state_ on CL/F	0.617	5.9%
θ_amisulpride_ on CL/F	0.392	17.8%
θ_amisulpride_ on CL_M_/F	0.593	28.7%
Interindividual variability, %CV		
CL/F	0.219	13.4%
*V*/F	0.106	35.2%
CL_M_/F	0.156	19.4%
*V*_M_/F	1.38	39.3%
Residual variability, % CV		
Proportional error on VEN	0.123	8.5%
Proportional error on ODV	0.101	13.6%

CL/F, clearance of VEN; *V*/F, volume of distribution of VEN; CL_M_/F, clearance of ODV; *V*_M_/F, volume of distribution of ODV; K_a,_ absorption rate constant; F_P_, fraction of the absorbed dose of VEN, converted into ODV, in first-pass metabolism.

### 3.3 Model evaluation

**FIGURE 4:**
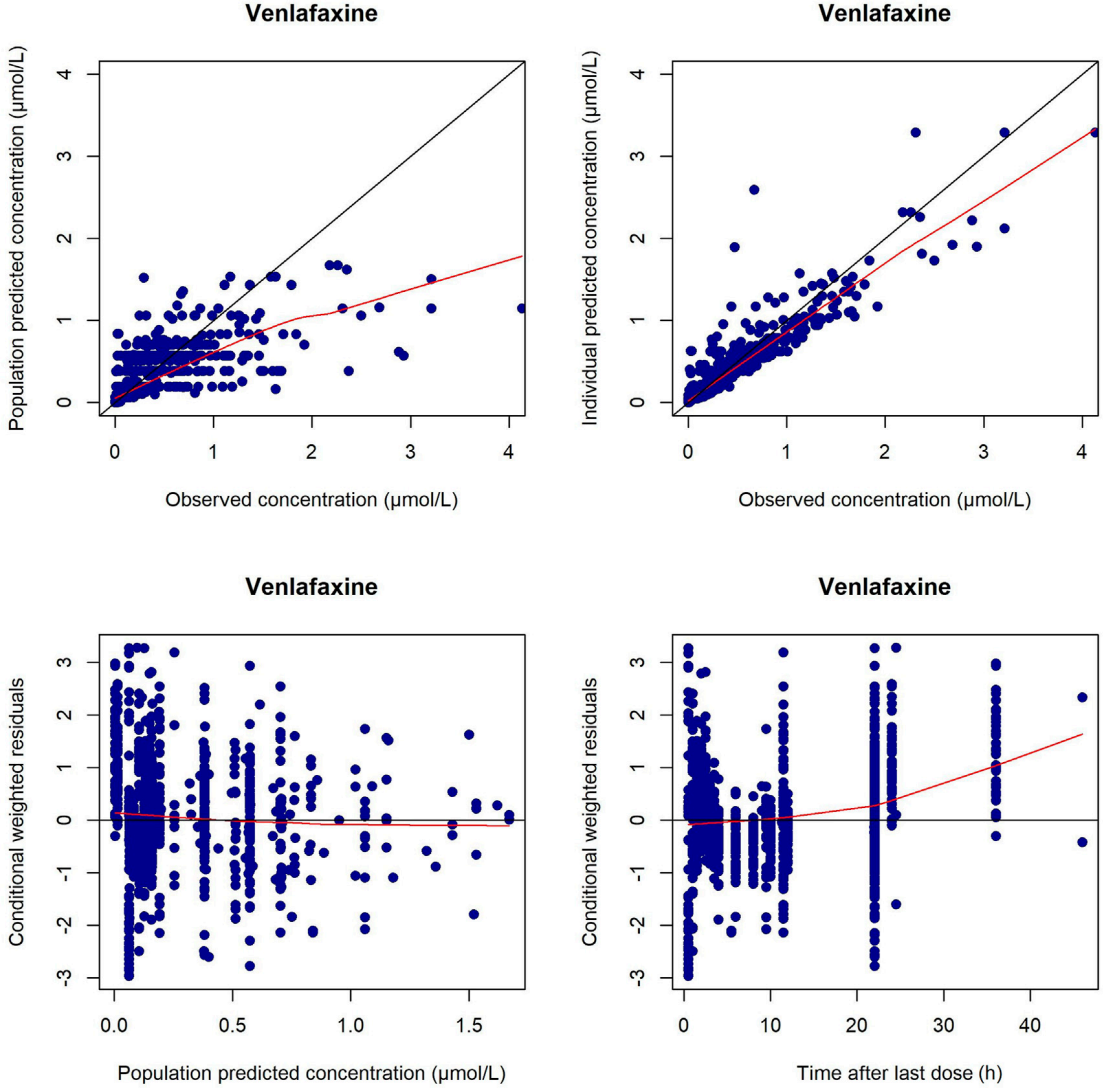
Diagnostic plots for VEN in the final PPK model. The left upper is the plot of the population-predicted concentrations versus the observed VEN concentrations; the right upper is the plot of the individual population-predicted concentrations versus the observed VEN concentrations; the left lower represents the conditional weighted residual error versus the population-predicted VEN concentrations; the right lower represents the conditional weighted residual error versus the time after last dose.

**FIGURE 5:**
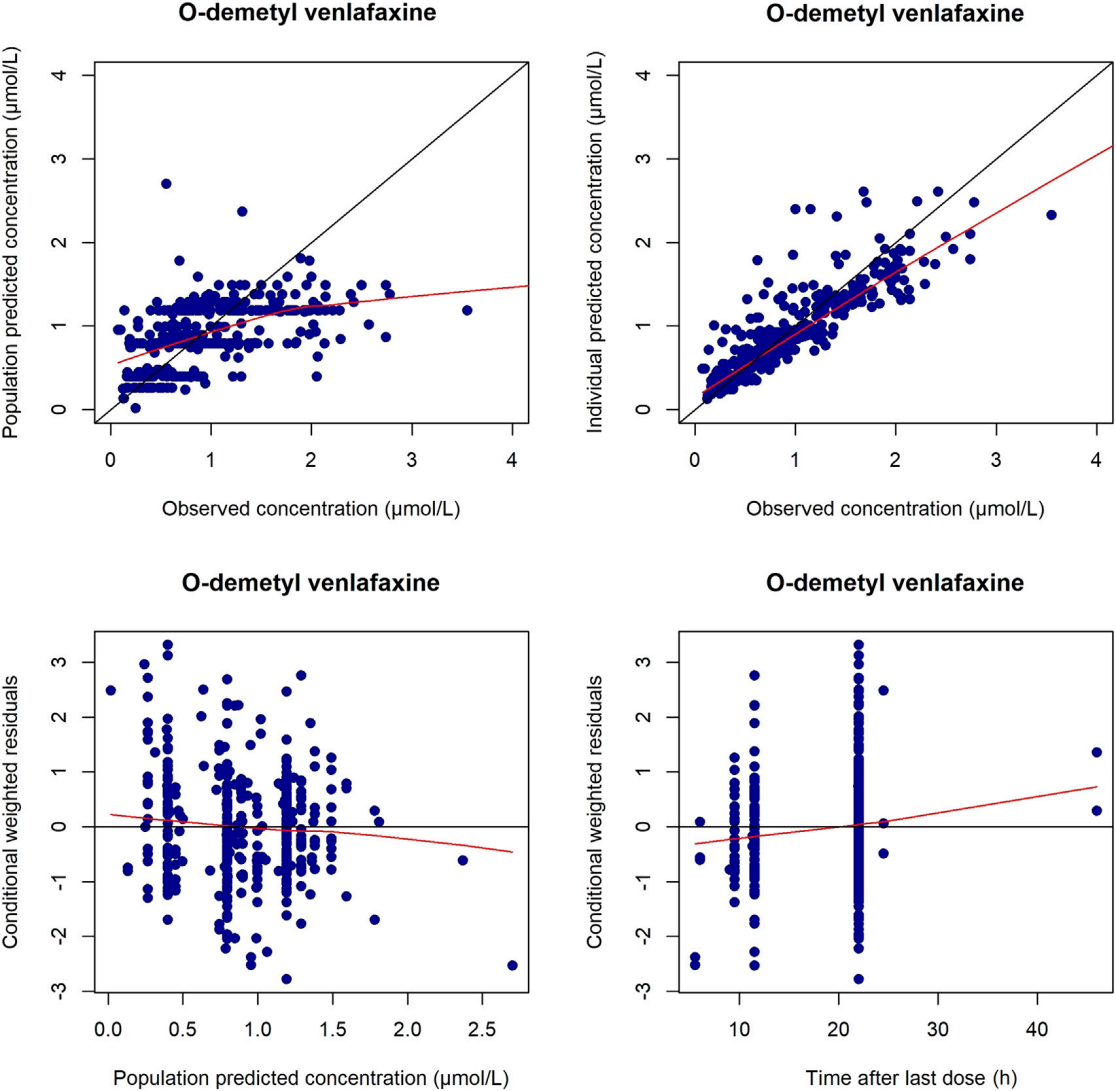
Diagnostic plots for ODV in the final PPK model. The left upper is the plot of the population-predicted concentrations versus the observed ODV concentrations; the right upper is the plot of the individual population-predicted concentrations versus the observed ODV concentrations; the left lower represents the conditional weighted residual error versus the population-predicted ODV concentrations; the right lower represents the conditional weighted residual error versus the time after last dose.

**FIGURE 6:**
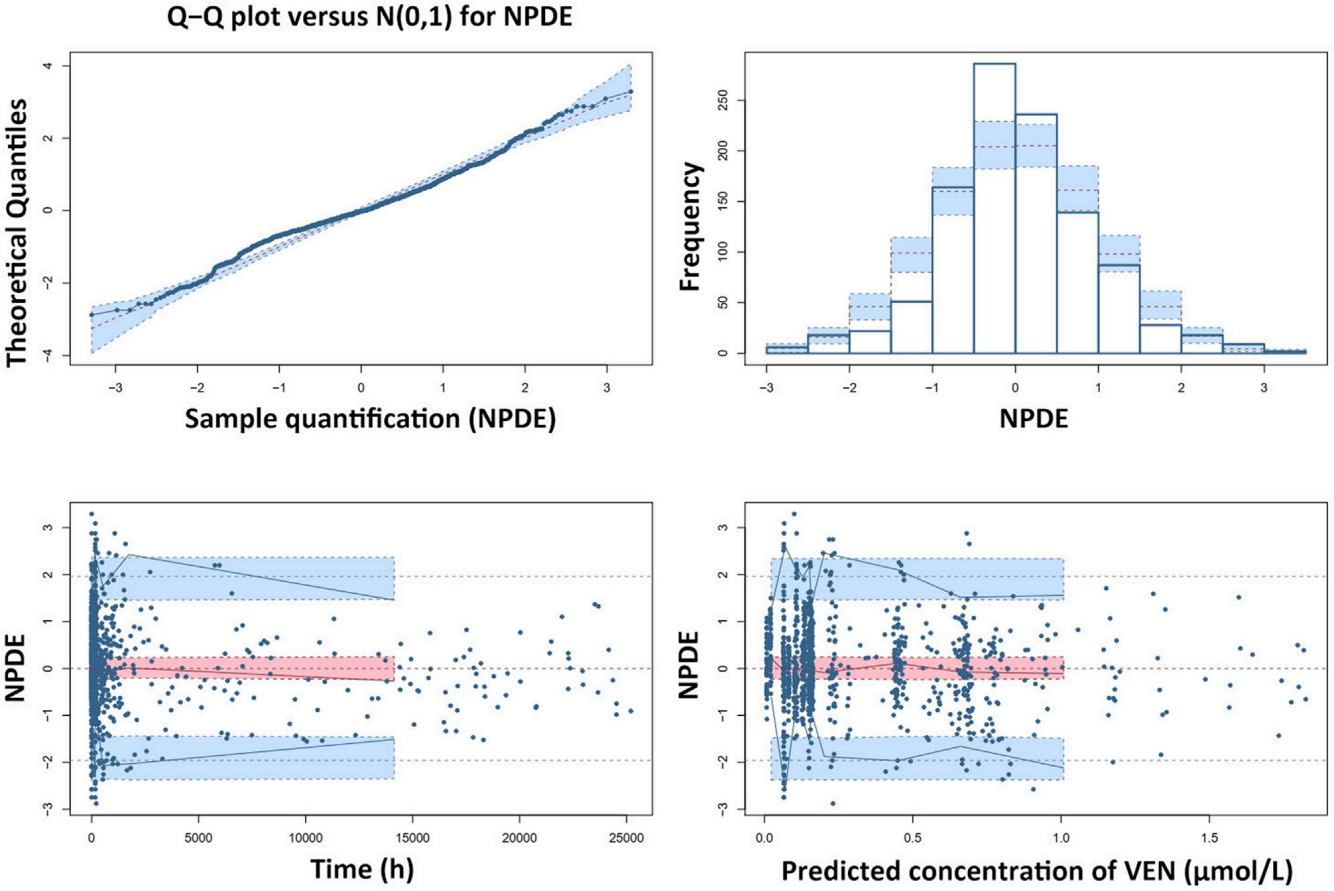
Results of the NPDE analysis for VEN. The left upper plot is a QQ-plot for NPDE; the right upper plot is a histogram of the NPDE; the left lower and right lower plots represent the NPDE versus time and the NPDE versus the predicted concentrations of VEN, respectively.

**FIGURE 7:**
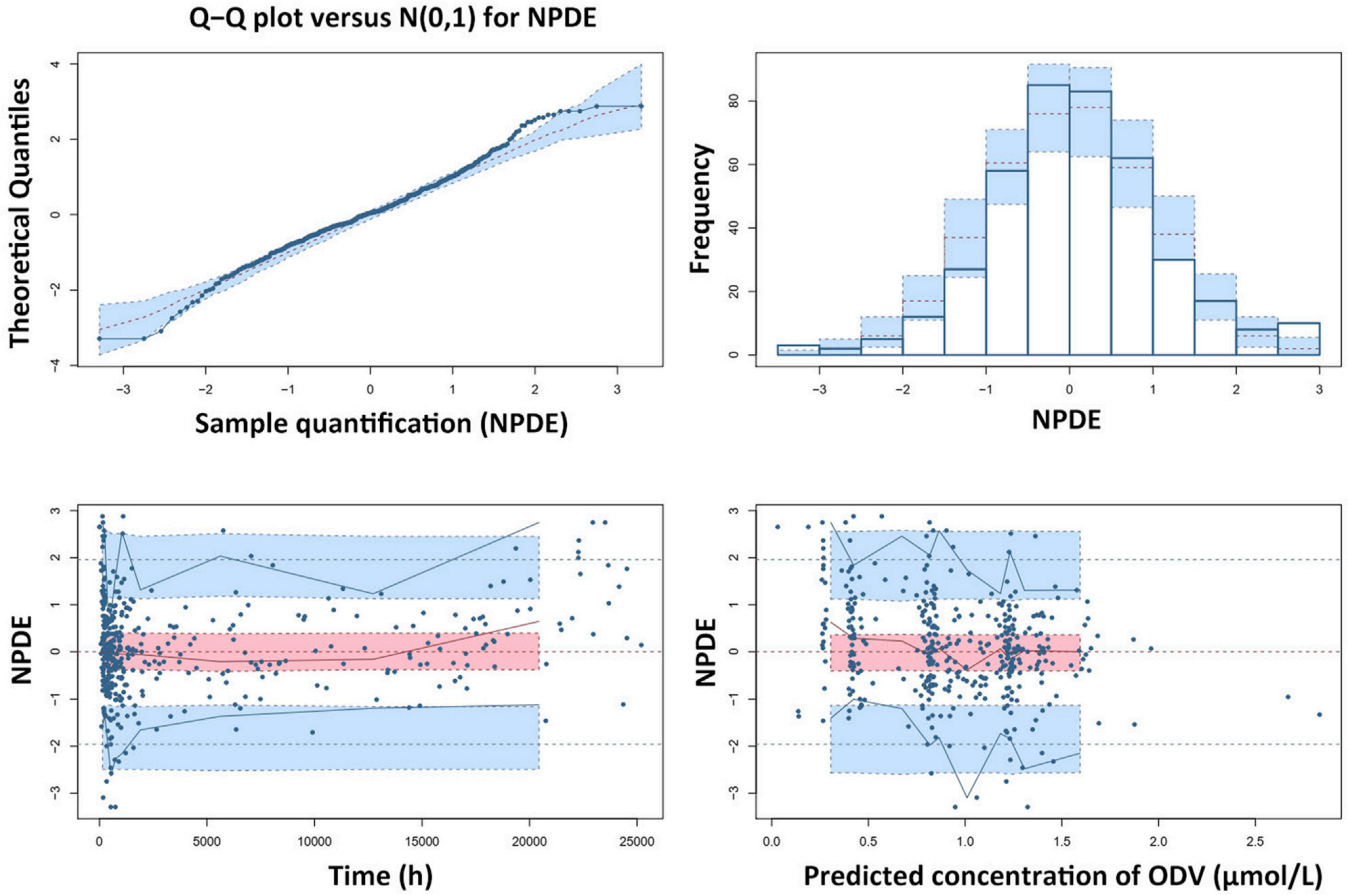
Results of the NPDE analysis for ODV. The left upper plot is a QQ-plot for NPDE; the right upper plot is a histogram of the NPDE; the left lower and right lower plots represent the NPDE versus time and the NPDE versus the predicted concentrations of ODV, respectively.

The GOF plots and NPDE prediction for VEN and ODV are shown in Figures 4–7. The implementation of covariates greatly improved the predictions for VEN and ODV. There was no trend of CWRES vs. PRED for VEN, but a slightly decreasing trend of CWRES vs. PRED for ODV. The CWRES increased with an increased time after last dose for VEN. For VEN and ODV, the NPDE agreed with the theoretical N (0, 1) distribution and density on the whole, which shows a good fit of the model to the individual data.

## 4 Discussion

VEN converts to ODV in gut and liver and exhibits high variations in concentrations. Considering the comparative pharmacological activity of ODV and VEN (Hiemke et al., 2018), there is a need to illustrate the pharmacokinetics of VEN and ODV simultaneously. However, to the best of our knowledge, no such study is available. In our study, the authors analyze intense samples including an absorption and elimination phase from a VEN PK study as well as sparse samples containing VEN and ODV concentrations from TDM. This study extends previous work by introducing a joint PPK model with pre-system metabolism and identifies two potential covariates affecting the clearance of VEN and ODV.

Because concentrations lower than the lower limit of quantification in the elimination phase were generally labeled as non-detectable and excluded from analysis, the estimated concentrations in the elimination phase could be higher than what they really were. In the GOF plots of the current model, the CWRES for VEN increased at 24 and 36 h post last dose was likely due to the undetectable concentrations in the elimination phase of study 1. Most conditional weighted residual values were between −3 and 3, and the NPDE was generally normally distributed. However, there existed bias in the estimation of extremely high or low concentrations. This was due to the nature of TDM, where most samples were trough concentrations, whose reference range was far from the extremely high and low concentrations. In general, the current model was still suitable for most samples in clinical practice.

This work integrated real-world TDM data relating to patients and bioequivalence study data from healthy volunteers. The estimated clearance of VEN and ODV in healthy male Chinese subjects were very close to published values of healthy male Caucasian and African subjects of similar age (Klamerus et al., 1992; Klamerus et al., 1996), while a small volume of distribution of VEN was found in Caucasian subjects (10.05 L/kg vs. 6.5 ± 1.5 L/kg (Klamerus et al., 1996)). The estimated half-life (5.4 h) and absorption time (1.6 h) of VEN in healthy subjects were generally comparable with those in previous reports (Klamerus et al., 1992; Bhatt et al., 2005; Wei et al., 2007; Reddy et al., 2013). These results suggest that the enzyme activity in different populations is not significantly different. There is no published pharmacokinetic data on VEN and ODV for patients in a naturalistic setting. The AGNP guideline gave quasimeasurements of clearance of VEN and its metabolites based on the deduction that the ratio of drug concentration to dose is an inverse value of oral clearance [Css = (D/t)/CL] (Reis et al., 2009). The estimated clearance was 72–75 L/h for VEN and 18–25 L/h for ODV (Hiemke et al., 2018), calculated by pooled data from healthy subjects and patients as indicated in reference. The estimated typical values of VEN and ODV clearance (81 L/h and 22 L/h) in our PPK study were close to previous reports. In our study, the morbid state caused a 62% reduction in clearance of VEN. The clearance of VEN in Chinese patients was close to that in poor Cytochrome P450 2D6 metabolizers (0.4 ± 0.14 L/h/kg (Preskorn et al., 2009)). Since VEN and ODV exerted a mild inhibitory effect on various CYP enzymes in particular CYP2D6 (Oganesian et al., 2009), it appeared to have a low potential for pharmacokinetic drug-drug interactions. But psychiatric patients usually need long-term treatments with psychotropic drugs, where the inhibitory effect of medications including VEN may enhance. Therefore, the altered clearance of VEN in psychiatric patients may be partly due to the long-term use of medications.

The impact of sex and age on the clearance of VEN and ODV has been investigated. In previous research (Wang et al., 2020), females tended to have smaller body weight and higher dose-corrected steady-state trough concentrations (Css/D) of VEN and VEN + ODV than male patients. In the current study, though clearance of VEN was higher in males than females (*p* < 0.001), this effect became insignificant in the final model. With respect to age, previous studies (Reis et al., 2002; Hansen et al., 2017; Wang et al., 2020) showed elderly patients had higher Css/D than younger patients. Though the clearance of VEN declined with age, the ΔOFV was small (*p* < 0.05) and this covariate was not included in the final model.

The influence of concomitant medications on the disposition of VEN and ODV was also evaluated. Though impacts of valproic acid on CL/F and CL_M_ (*p* < 0.05), quetiapine on *V*/F (*p* < 0.01), clozapine on CL_M_/F (*p* < 0.01), risperidone on *V*/F (*p* < 0.05) and amisulpride on CL/F and CL_M_/F (*p* < 0.001) were found at the beginning, only amisulpride was identified as a covariate for CL/F and CL_M_/F finally. The amisulpride caused a 39.2% reduction in clearance of VEN and a 59.3% increase in clearance of ODV. However, the underlying mechanism was unknown. Amisulpride was neither metabolized by rat liver microsomes (Schmitt et al., 2006) nor altered the enzyme activity, thus the enzyme was not supposed to be the reason for potential drug-drug interaction. On the other hand, VEN and to a lesser extent ODV, induced the expression of P-glycoprotein (P-gp) (Bachmeier et al., 2013). Since amisulpride was also a substrate of P-gp (Schmitt et al., 2006), it seems reasonable to speculate the pharmacokinetic interaction was related to transporters. In addition, both VEN and amisulpride prolonged the QT interval (Wenzel-Seifert et al., 2011) and might increase the likelihood to cause cardiac adverse events (Elsayed et al., 2021). It should be cautious for physicians to prescribe these drugs concomitantly.

There were several limitations in our study. First, the best-fit models for intravenous VEN and ODV were three-compartment model and two-compartment model, respectively, according to pharmacokinetics after IV infusions of VEN and ODV (Lin et al., 2016). But in the current study, a one-compartment model was used to describe the pharmacokinetics of VEN and ODV. There are several reasons: this study was constituted mainly of TDM data, which were usually sparse trough concentrations; VEN undergoes extensive metabolism and converts to ODV in the gut and liver; the use of one-compartment model can simplify the analysis process. Second, the K_a_ of VEN varied from 0.08 to 1.31 h^−1^ in previous reports (Taft et al., 1997; Chen et al., 2014; Xie et al., 2020). We conducted a two-compartment PPK model for VEN (not published) and estimated the typical value of K_a_ to be 0.63 h^−1^. Then the K_a_ in the current study was fixed at 0.63 h^−1^. We also built PPK models with different K_a_ (0.5- and 2-fold) and found mild influence on estimations of pharmacokinetic parameters. Third, the number of samples in subgroups was relatively small. The impact of comedications needs to be verified in a larger population. In addition, comedication like doxepin (Paulzen et al., 2018a) was identified to increase concentrations of VEN and VEN + ODV in patients in other research, but this concomitant medication was rare and therefore not discussed in our study. Fourth, the underlying mechanisms of morbidity state and the concomitant use of amisulpride on clearance are unknown and need to be further explored. Finally, genotypes or metabolizer status of CYP2D6 and CYP2C9, which could contribute to the efficacy (Ahmed et al., 2019), are not discussed here, due to the limited available data. Additional study may be needed for the quantification of mixed effects about enzyme phenotypes and comedications.

## 5 Conclusion

In the present study, a joint PPK model was developed for VEN and ODV to describe the complex pharmacokinetics of both bioactive moieties in humans. The pre-system metabolism was modeled through the incorporation of a rate constant for first-order conversion. The morbid state and concomitant amisulpride were identified as two significant covariates affecting the clearance of VEN and ODV, but further studies on the mechanisms are needed to generalize the result. These findings may account for some of the variability in exposure. This model may contribute to the precision medication in clinical practice and may inspire other drugs with pre-system metabolism.

## Data Availability

The original contributions presented in the study are included in the article/Supplementary Materials, further inquiries can be directed to the corresponding authors.
